# Modulation of Nod-like Receptor Expression in the Thymus during Early Pregnancy in Ewes

**DOI:** 10.3390/vaccines10122128

**Published:** 2022-12-13

**Authors:** Leying Zhang, Yuanjing Li, Zhenyang Zhao, Jiabao Cai, Shuxin Zhao, Ling Yang

**Affiliations:** Department of Animal Science, School of Life Sciences and Food Engineering, Hebei University of Engineering, No. 19 Taiji Road, Handan 056038, China

**Keywords:** nucleotide-binding oligomerization domain receptor, pregnancy, sheep, thymus

## Abstract

Nucleotide-binding oligomerization domain receptors (NOD-like receptors, NLRs) are involved in modulating the innate immune responses of the trophoblast and the placenta in normal pregnancy. The thymus participates in regulation of innate and adaptive immune responses. However, it is unclear whether expression of NLR is modulated in the maternal thymus during early pregnancy. In this study, thymuses were sampled at day 16 of the estrous cycle, and at days 13, 16 and 25 of gestation (*n* = 6 for each group) from ewes after slaughter. Different stages were chosen because the maternal thymus was under the different effects of interferon-tau and/or progesterone or not. RT-qPCR, Western blot and immunohistochemistry analysis were used to analyze the expression of the NLR family, including NOD1; NOD2; major histocompatibility complex class II transactivator (CIITA); NLR family apoptosis inhibitory protein (NAIP); nucleotide-binding oligomerization domain and Leucine-rich repeat and Pyrin domain containing protein 1 (NLRP1), NLRP3 and NLRP7. The results showed that expression level of NOD1 was changed with the pregnancy stages, and expression levels of NOD2, CIITA, NAIP, NLRP1, NLRP3 and NLRP7 mRNA and proteins were peaked at day 13 of pregnancy. The levels of NOD2 and CIITA were increased during early pregnancy. The stainings for NOD2 and NLRP7 proteins were located in epithelial reticular cells, capillaries and thymic corpuscles. In summary, pregnancy stages changed expression of NLR family in the maternal thymus, which may be related to the modulation of maternal thymic immune responses, and beneficial for normal pregnancy in sheep.

## 1. Introduction

The nucleotide-binding oligomerization domain receptor (NOD-like receptors, NLR) family mediates the initial innate immune responses to cellular injury and stress, and several NLRs are involved in regulation of a variety of signaling pathways [1]. NOD1 and NOD2 are expressed in B and T cells and play critical roles in development, maintenance, regulation and activation of the innate and adaptive immune system [2]. Major histocompatibility complex (MHC) class II transactivator (CIITA) is critical for MHC II molecules in immune responses and also expressed in macrophages to participate in immune responses [3]. NLRPs (nucleotide-binding oligomerization domain, Leucine-rich repeat and Pyrin domain containing proteins) are implicated in innate immunity [4], and recessive mutation on NLRP7 is related with reproductive disorders in humans [5]. Interferon regulatory factor 8 is a master transcription factor for immune cell development and governs the constitutive expression of genes encoding for NLR family apoptosis inhibitory protein (NAIP) that is critical for the innate immune sensing in humans [6]. NLRs are expressed in the trophoblast and the placenta and involved in regulating the innate immune responses of these tissues in normal and pathological pregnancies [7].

Balance of the immune response is required for successful implantation, placental development and fetal growth in pregnancy and excessive activation of the immune system increases risk of adverse pregnancy outcomes [8]. Interferon-tau (IFNT) is involved in the recognition of pregnancy and maintenance of corpus luteum (CL) function and also has paracrine and endocrine actions in modulating the innate immune system to avoid conceptus rejection in ruminants [9]. There is an immune tolerance in maternal immune system during pregnancy, which is induced locally and systemically by IFNT and progesterone, pregnancy associate glycoproteins and early pregnancy factor in ruminants [10]. IFNT has effects on the embryo, endometrial cells, several extrauterine tissues and cells and participates in the interaction between early embryo and maternal immune system to achieve the establishment of pregnancy in ruminants [11]. IFNT and progesterone have systemic effects on immune organs, which modulate expression of interferon-stimulated genes (ISGs) and progesterone receptors in the ovine bone marrow [12,13], the thymus [14,15], the spleen [16,17,18] and lymph nodes [19,20,21] during early pregnancy.

The lymphoid system is intimately related to immunological processes, and the thymus in the lymphoid system is involved in lymphocyte production and ensuring the normal development of immunological faculty through the bloodstream [22]. The peripheral B cells circulate through the thymus that is involved in mediate negative selection of B cells, and related with establishment of central immune tolerance in humans and mice [23]. Osteoclast differentiation receptor couples female sex hormones to participate in development of thymic Treg cells, which are involved in marked changes of the thymus during pregnancy in mice [24]. There is an increase in thymic output of conventional and regulatory T cells, which contribute to immunological tolerance toward the semiallogeneic fetus during pregnancy in humans [25]. It has been reported that there is an upregulation of progesterone receptor isoform (60 kDa), progesterone-induced blocking factor variant (62 kDa), tumor necrosis factor (TNF) beta, gonadotropin releasing hormone, melatonin receptors, interleukin-10 (IL-10) and prolactin and its receptor, and the expression levels of cyclooxygenase 2, prostaglandin E synthase, aldo-keto reductase family 1, member B1, interferon-γ, IL-5, IL-6 and cluster of differentiation 4 (CD4) are changed in the ovine thymus during early pregnancy [14,26,27,28,29,30]. In addition, early pregnancy modulates expression of the Toll-like receptors (TLRs); TNF receptor associated factor 6; IL-1 receptor associated kinase 1; myeloid differentiation primary response gene 88 [31] and the complement components C1q, C1r, C1s, C2, C3, C4a, C5b and C9 in the ovine thymus [32].

It was supposed that early pregnancy had effects on the expression of NLR family in the ovine thymus. The objective of this study was to explore the expression of NOD1, NOD2, CIITA, NAIP, NLRP1, NLRP3 and NLRP7 in the maternal thymus during early pregnancy in sheep, and the results may be useful for understanding the additional function of NLR family in maternal thymic immunomodulation during early pregnancy in ruminants.

## 2. Materials and Methods

### 2.1. Animals and Experimental Design

The study began during the normal breeding season in September and finished in January. Non-pregnant ewes (Small-tail Han sheep) with approximately 2 years of age and similar genetic backgrounds were housed at a local farm in the Hebei Province, China. The controlled internal drug release (CIDR) device was used for estrus synchronization, and the ewes were randomly divided into four groups based on the body condition score (*n* = 6 for each group). After removal of the CIDR, the ewes were checked twice daily for estrus and then mated to either intact or vasectomized rams at estrus (day 0). Thymic tissues were collected at days 13, 16 and 25 of pregnancy and day 16 of the estrous cycle after the females were euthanized by captive bolt and exsanguination. Pregnancy was validated by the presence of a normal conceptus in the uterine lumen. Different stages were chosen because the maternal thymus was under the different effects of IFNT and/or progesterone or not [28]. Longitudinal cross-sections of the thymuses were immersion fixed in fresh 4% buffered paraformaldehyde for subsequent immunohistochemical analysis. In addition, Transverse pieces of the thymuses were snap-frozen in liquid nitrogen (−196 °C) for subsequent mRNA and protein analysis.

### 2.2. RNA Extraction and RT-qPCR Assay

Total RNA was extracted from the thymic transverse pieces using TRNzol Universal Reagent (DP424; Tiangen Biotech Co., Ltd., Beijing, China) according to the manufacturer’s instructions. The total RNA (approximately 1 µg) was reverse transcribed into cDNA using a FastQuant RT kit with gDNase (KR106; Tiangen Biotech). The specified primers (Table 1) were designed based on the sequences in the NCBI database (http://www.ncbi.nlm.nih.gov/ (accessed on 2 May 2021)) for the ovine NOD1, NOD2, CIITA, NAIP, NLRP1, NLRP3 and NLRP7 genes and synthesized by Shanghai Sangon Biotech Co., Ltd. (Shanghai, China). The reactions were carried out at 95 °C for 10 min, followed by 40 cycles of denaturation (95 °C for 10 s), annealing (59 to 62 °C for 20 s) and extension (72 °C for 25 s) followed by one cycle of final extension (72 °C for 7 min). The annealing temperatures were 60.5 °C for NOD1 and CIITA or 62 °C for NOD2 or 59.5 °C for NAIP or 60 °C for NALP1 or 59 °C for NLRP3 or 61 °C for NLRP7. Quantitative PCR was performed using a SuperReal PreMix Plus kit (Tiangen Biotech Co., Ltd., Beijing, China) in a total volume of 20 μL in triplicate in a CFX96 real-time PCR system (Bio-Rad Laboratories, Inc., Hercules, CA, USA) A reference gene (glyceraldehyde phosphate dehydrogenase gene, GAPDH) was used for normalization, and analyzed in parallel in all target genes. The 2^−ΔΔCt^ analysis method was used to calculate the relative values for the target genes [33], and mean cycle threshold (Ct) values from the group of day 16 of the estrous cycle were used to normalize the relative levels of mRNA transcripts.

### 2.3. Western Blot Analysis

Total proteins were isolated from thymic tissues of all ewes by RIPA lysis buffer supplemented with phosphatase and protease inhibitor on ice. Protein concentrations were determined using a bicinchoninic acid assay kit (Tiangen Biotech). Equal amounts of total protein (10 μg per well) were separated using 12% SDS-PAGE gels, followed by transfer electrophoretically onto methanol-activated polyvinyl difluoride membranes (Millipore, Bedford, MA, USA) for immunoblotting. The membranes were blocked in 5% (*w*/*v*) nonfat milk at 4 °C overnight, followed by incubation with primary antibodies in a 1:1000 dilution at 4 °C overnight. The primary antibodies include a mouse anti-NOD1 monoclonal antibody (Santa Cruz Biotechnology, Santa Cruz, CA, USA, sc-398696), a mouse anti-NOD2 monoclonal antibody (Santa Cruz Biotechnology, sc-56168), a mouse anti-CIITA monoclonal antibody (Santa Cruz Biotechnology, sc-13556), a rabbit anti-NAIP polyclonal antibody (Abcam, Cambridge, UK, ab25968), a mouse anti-NLRP1 monoclonal antibody (Santa Cruz Biotechnology, sc-390133), a mouse anti-NLRP3 monoclonal antibody (Santa Cruz Biotechnology, sc-134306) and a mouse anti-NLRP7 monoclonal antibody (Santa Cruz Biotechnology, sc-377190). The antibodies were validated by ovine proteins and suitable for sheep. The membranes were incubated with a secondary antibody (anti-mouse IgG-HRP BL001A or anti-rabbit IgG-HRP BL003A) in a 1:10000 dilution after washed in Tris-buffered saline containing 0.05% Tween-20. The primary antibodies were detected using an ECL Western blot detection kit (Tiangen Biotech) with X-ray films (Fujifilm, Tokio, Japan). The optical densitometry values of the blots were analyzed using Quantity One V452 (Bio-Rad Laboratories, Hercules, CA, USA), and GAPDH-horseradish peroxidase antibody (Santa Cruz Biotechnology, sc-47724, 1:1000) was used as the loading control.

### 2.4. Immunohistochemistry Analysis

Immunohistochemical procedures were described previously by Zhang et al. [32]. Briefly, thymic tissues were sectioned at 5 μm thick, mounted onto glass slides, deparaffinized and rehydrated in a series of xylene to ethanol. Some thymic sections were stained by hematoxylin and eosin. Heat-induced antigen retrieval was performed in boiling citrate solution, and thymic sections were treated with blocking buffer for blocking endogenous peroxidase activity. After the tissues were incubated with 5% normal goat serum in PBS for blocking nonspecific binding sites, they were incubated with primary antibodies for NOD2 (1:200 dilution; sc-56168, Santa Cruz Biotechnology) or for NLRP7 (1:200 dilution; sc-377190, Santa Cruz Biotechnology) overnight at 4 °C with shaking. Primary antibodies were detected using a secondary antibody (1:1000 dilution; anti-mouse IgG-HRP, BL001A) and a DAB kit (Tiangen Biotech), according to the manufacturer’s instructions. Sections were then counterstained with hematoxylin. Control sections were incubated with an antiserum-specific isotype at the same protein concentration in place of primary antibody. Images of thymic tissues were generated using a light microscope (Nikon Eclipse E800, Tokyo, Japan) equipped with a DP12 digital camera. Finally, the images were analyzed independently by 4 experienced observers with the similar observations for all cases. The immunostaining intensities of the thymic samples from different ewes were examined through the images in a blinded manner. Staining intensities for NOD2 or NLRP7 protein were calculated by assigning an immunoreactive intensity of a scale of 0 to 3, as described in a previous report [32].

### 2.5. Statistical Analysis

Statistical analysis was performed using least-squares ANOVA in Mixed and General Linear Model procedures of the Statistical Analysis System (Version 9.2; SAS Institute, Cary, NC, USA). Day and status (cyclic or pregnant) and interaction between day and status on the expression of mRNA and proteins in the maternal thymus were tested using repeated measures for a multivariate analysis of variance. All data were expressed as means ± standard deviation, and comparisons of means were made using the Duncan method. *p* values less than 0.05 was considered significant.

## 3. Results

### 3.1. Relative Expression Levels of NOD1, NOD2, CIITA, NAIP, NLRP1, NLRP3 and NLRP7 mRNA in the Thymus

**Figure 1:**
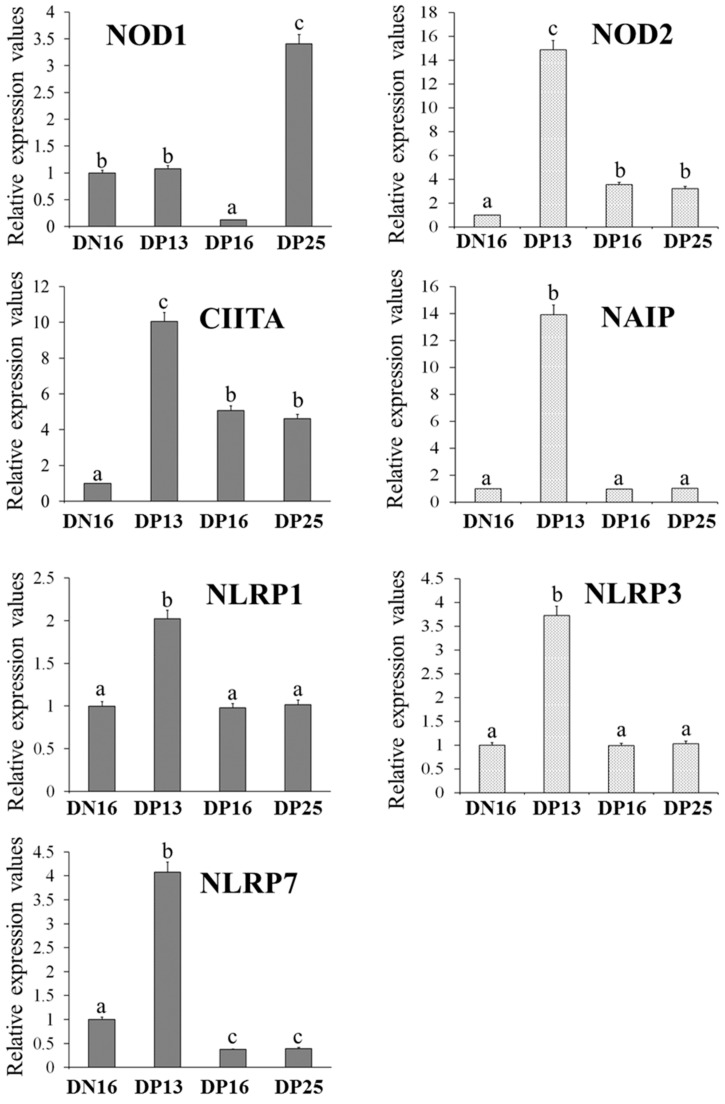
Relative expression values of NOD1, NOD2, CIITA, NAIP, NLRP1, NLRP3 and NLRP7 mRNA in ovine thymus measured by quantitative real-time PCR. Note: DN16 = day 16 of the estrous cycle; DP13 = day 13 of pregnancy; DP16 = day 16 of pregnancy; DP25 = day 25 of pregnancy. Significant differences (*p* < 0.05) are indicated by different letters within same color column.

(A) There was a downregulation of the relative mRNA level of NOD1 mRNA at day 16 of pregnancy but an upregulation at day 25 of pregnancy comparing to day 16 of the estrous cycle and day 13 of pregnancy (*p* < 0.05; Figure 1).

(B) The relative mRNA levels of NOD2, CIITA, NAIP, NLRP1, NLRP3 and NLRP7 were peaked at day 13 of pregnancy, and the levels of NOD2 and CIITA were higher at days 16 and 25 of pregnancy than day 16 of the estrous cycle (*p* < 0.05; Figure 1).

(C) There was no significant difference among the expression levels of NAIP, NLRP1 and NLRP3 at day 16 of the estrous cycle and days 16 and 25 of pregnancy (*p* > 0.05; Figure 1), and the NLRP7 level was lower at days 16 and 25 of pregnancy than day 16 of the estrous cycle (*p* < 0.05; Figure 1).

### 3.2. Expression of the NOD1, NOD2, CIITA, NAIP, NLRP1, NLRP3 and NLRP7 Proteins in the Thymus

**Figure 2:**
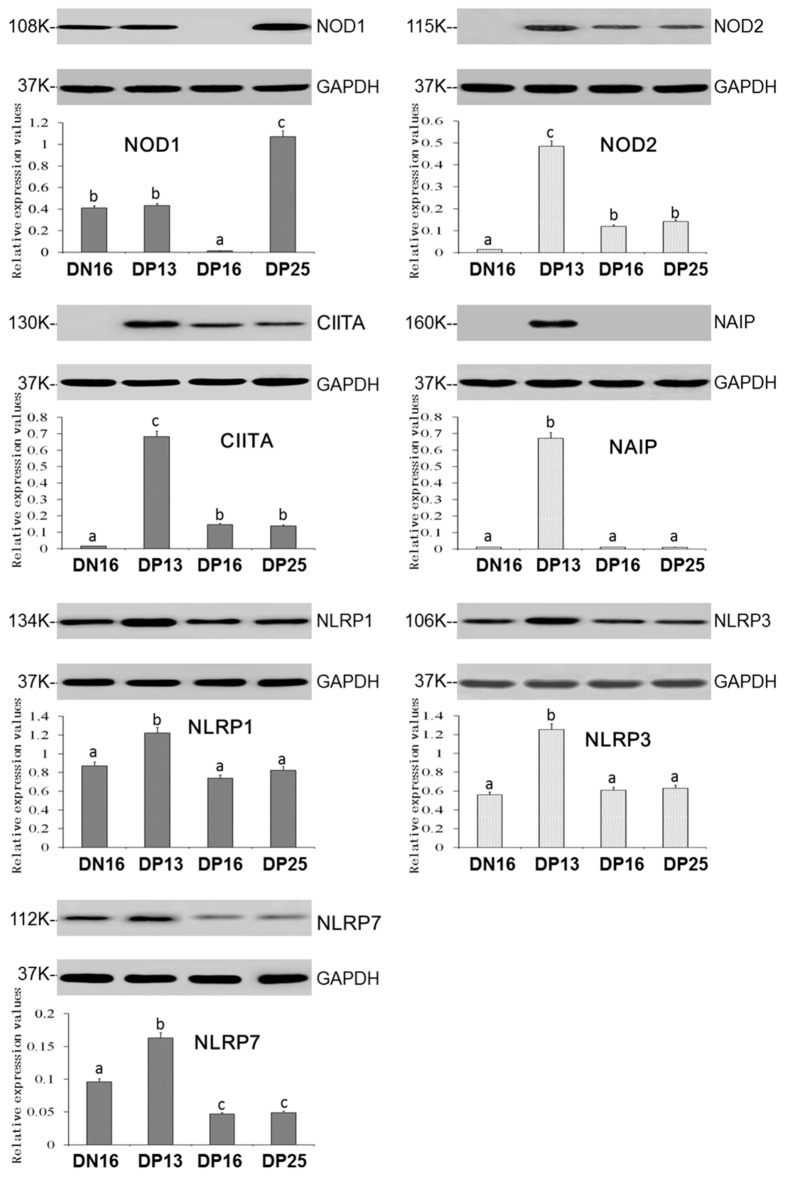
Expression of NOD1, NOD2, CIITA, NAIP, NLRP1, NLRP3 and NLRP7 proteins in the ovine thymus analyzed by Western blot. Note: DN16 = day 16 of the estrous cycle; DP13 = day 13 of pregnancy; DP16 = day 16 of pregnancy; DP25 = day 25 of pregnancy. Significant differences (*p* < 0.05) are indicated by different superscript letters within the same color columns.

(A) NOD1 protein level was decreased at day 16 of pregnancy but increased at day 25 of pregnancy compared to day 16 of the estrous cycle and day 13 of pregnancy (*p* < 0.05; Figure 2).

(B) The protein levels of NOD2, CIITA, NAIP, NLRP1, NLRP3 and NLRP7 were the highest at day 13 of pregnancy among the four groups, and NOD2 and CIITA proteins were present at days 16 and 25 of pregnancy but undetected at day 16 of the estrous cycle (Figure 2).

(C) There was no significant difference among the protein levels of NLRP1 and NLRP3 at day 16 of the estrous cycle and days 16 and 25 of pregnancy (*p* > 0.05; Figure 1).

(D) NAIP protein was undetected at day 16 of the estrous cycle and days 16 and 25 of pregnancy (Figure 2), and the NLRP7 protein level was higher at day 16 of the estrous cycle than days 16 and 25 of pregnancy (*p* < 0.05; Figure 2).

### 3.3. Immunohistochemistry for NOD2 and NLRP7 Proteins in the Thymus

**Figure 3:**
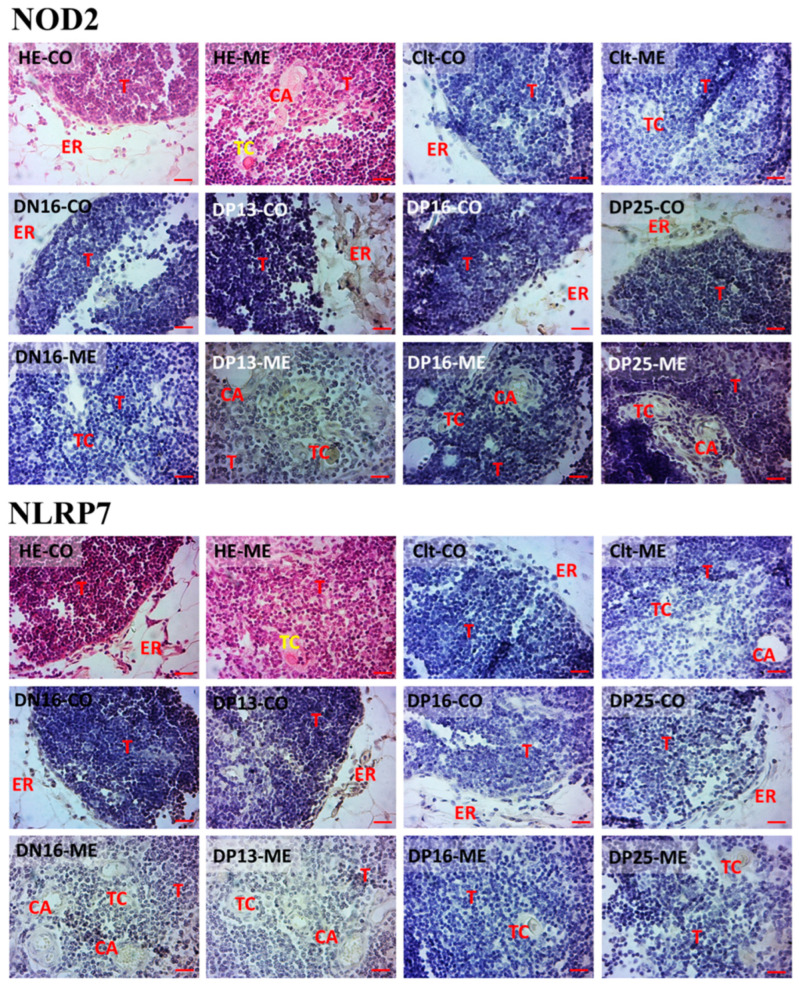
Representative immunohistochemical localization of NOD2 and NLRP7 proteins in the ovine thymus. The thymus is divided into the cortex (CO) and the medulla (ME). Note: HE = stained by hematoxylin and eosin; Ctl = negative control; DN16 = day 16 of the estrous cycle; DP13 = day 13 of pregnancy; DP16 = day 16 of pregnancy; DP25 = day 25 of pregnancy; T = thymocyte; ER = epithelial reticular cell; CA = capillary; TC = thymic corpuscle. Bar = 20 µm.

(A) The NOD2 and NLRP7 proteins were mainly located in epithelial reticular cells, capillaries and thymic corpuscles (Figure 3).

(B) The staining intensities for the NOD2 protein were 0, 0, 3, 2 and 2, while the NLRP7 protein were 0, 2, 2, 1 and 1 for the negative control, the thymuses from day 16 of the estrous cycle and thymuses from days 13, 16, and 25 of pregnancy, respectively (Figure 3). The staining intensity was as follows: 0 = negative, 1 = weak, 2 = strong and 3 = stronger.

## 4. Discussion

NOD1 modulates multiple pathways to participate in a variety of cellular responses, including inflammatory responses, and plays an essential role in maintaining tissue and immune homeostasis [2]. There is a lower level of NOD1 in the decidual stromal cells derived from unexplained recurrent pregnancy loss than normal early trimester pregnancy, and NOD1 is involved in maintaining pregnancy via modulating immune responses [34]. NOD1 has effects on interaction between decidual stromal cells and trophoblast, which is necessary for trophoblast invasion during the early trimester in humans [35]. NOD1 is expressed in human first-trimester trophoblasts, but the administration of bacterial γ-D-glutamyl-meso-diaminopimelic acid, one of the NOD1 agonist, induces preterm delivery of pregnant mice on embryonic day 14.5 [36]. Increasing the level of NOD1 protein results in a higher level of inflammatory mediator that contributes to the development of intrauterine growth restriction [37]. In this study, the expression of NOD1 was downregulated on day 16 of pregnancy and then upregulated on day 25 of pregnancy. Therefore, the downregulation of NOD1 on day 16 of pregnancy may be helpful for the attenuated immune responses, and the slightly upregulation may be related with the immune regulation of pregnancy.

NOD2 is an intracellular sensor for small peptides, plays key roles in innate immune responses and also cross-talks with TLRs to participate in the development of autoimmunity [38]. Normal decidual stromal cells from the first trimester express NOD2 that has roles in the immunologic protection at the maternal-fetal interface during the first trimester of pregnancy in humans [39]. There is a lower level of NOD2 in decidual stromal cells from patients with unexplained recurrent spontaneous abortion than the normal pregnancy group, suggesting that a lower level of NOD2 is related to complex autoimmune disorder in humans [40]. NOD2 is expressed in the first trimester placental villi and localized to trophoblast cells that generate a proinflammatory cytokine response after treatment of a NOD2 ligand, muramyl dipeptide, suggesting that NOD2 can recognize and respond to invasive intracellular pathogens or foreign antigen [41]. There are thymocyte maturation defects in commensal bacteria-free animals, but exogenous NOD2 can enhance thymocyte maturation in culture [42]. Our results revealed that NOD2 mRNA and protein were upregulated in the maternal thymus during early pregnancy, and NOD2 protein was located in epithelial reticular cells, capillaries and thymic corpuscles. Therefore, the upregulation of NOD2 may be associated with modulation of the maternal thymic immune responses and helpful for the establishment and maintenance of pregnancy.

CIITA is the master control factor for the expression of MHC class II (MHCII) genes and expressed in most cell types, including trophoblasts and immune cells, to participate in immune surveillance [43]. Interferon-γ from conceptuses induces the expression of CIITA in a cell type- and pregnancy status-specific manner in the endometrium, which is involved in the interactions between the maternal uterine endometrium and the implanting conceptus during the implantation period in pigs [44]. The difference in the transcriptomic profiles between somatic cell nuclear transfer embryos and in vivo- and in vitro-derived embryos is the expression of CIITA in inner cell mass side samples of the embryos, which have biological function in preimplantation embryos in cattle [45]. CIITA is expressed in peripheral CD4 and CD8 T cells and thymocytes, which are involved in T cell development and differentiation and the production of Th2 type cytokines [46]. Early pregnancy enhances the expression of Th2 type cytokines, including IL-5, IL-6 and IL-10, in maternal thymus in sheep, and IL-6 protein is limited to the stromal cells, capillaries and thymic corpuscles [26]. In this study, early pregnancy enhanced the expression of CIITA in the maternal thymus, with the highest expression level at day 13 of pregnancy, suggesting that the upregulation of CIITA may be related with the maternal thymic immune regulation and useful for the embryo implantation in sheep.

NAIP activates caspase-1, which cleaves the pro-form of the inflammatory immune mediators and initiates NLR-mediated innate immunity [47]. Activation of NAIP/NLR-family card-containing protein 4 inflammasome triggers the canonical inflammasome outcomes of cytokine cleavage, which leads to a strong autoinflammatory response [48]. NAIP is an immune detector protein of the innate immune system, and its activation results in assembly of a large multiprotein complex that initiates innate immune responses through activating the caspase-1 protease [49]. Long noncoding RNA AK002210 improves the proliferation, migration and invasion of trophoblast cells through the regulation of NAIP expression, which is implicated in the modulation of phenotype of the trophoblast cell, as well as the progression of preeclampsia [50]. It is found in this study that NAIP protein was only expressed at day 13 of pregnancy, and this expression may be related to the modulation of maternal thymic functions, and the no undetected NAIP protein may be helpful for pregnancy maintenance.

NLRP1 acts as an immune sensor to recognize and respond to pathogen-associated activities and triggers an innate immune response via a ‘functional degradation’ model [51]. Altered homeostasis leads to the activation of NLRP1 that is present in several immune and nonimmune cell types and tissues, which are involved in inflammatory process and adaptive immunity response [52]. There is a higher endogenous activation of NLRP1/NLRP3 inflammasomes in the monocytes from preeclamptic women than normotensive pregnant women, but Silibinin can inhibit NLRP1/NLRP3 inflammasomes in monocytes from pregnant women with preeclampsia [53]. A higher level of NLRP1 in placental trophoblasts induces abortion in a rat oxidative stress model, which is associated with the pathogenesis of pathological pregnancy and adverse pregnancy [54]. The thymus expresses NLRP1 that can sense exogenous and endogenous pathogen-associated molecular patterns and is related to inflammation and innate immune responses [47]. Our data showed that early pregnancy induced expression of NLRP1 mRNA and protein at days 13 and 16 of pregnancy but then declined at day 25 of pregnancy. Therefore, the upregulation of NLRP1 may be related to the modulation of maternal thymic immune functions, but the downregulation of NLRP1 may be essential for pregnancy establishment.

The NLRP3 inflammasome is expressed in many cells, including macrophages, neutrophils, monocytes and dendritic cells, and also expressed in nonimmune cells, including endothelial, hepatocytes and vascular smooth muscle cells [55,56]. Activation of the NLRP3 inflammasome is associated with a physiological endoplasmic reticulum stress and unfolded protein response, which are implicated in the decidualization and embryo implantation [57]. Uric acid enhances trophoblast IL-1β production via activation of the NLRP3 inflammasome and production of IL-1β, which are implicated in the pathogenesis of preeclampsia and adverse pregnancy outcome [58]. IL-1β is involved in endothelial cell activation induced by necrotic trophoblastic debris, and NALP3 inflammasome is related to the production of IL-1β but not involved in the endothelial cell activation [59]. The thymus expressed NALP3 in constitutive status, which triggers innate immune responses to guard important adaptive immunity in developing organs [60]. Our results revealed that NLRP3 expression level was upregulated at day 13 of pregnancy but returned to normal level at days 16 and 25 of pregnancy. Therefore, the upregulation of NLRP3 may be associated with the modulation of maternal thymic innate immune and adaptive immune responses during early pregnancy.

NLRP7 has effects on inflammasome responses that are essential for homeostasis and host defense, but NLRP7 mutations result in molar pregnancy in humans [61]. NLRP7 is involved in immune tolerance via the regulation of key immune tolerance-associated factors and also improves trophoblast proliferation and decreases their differentiation during normal pregnancy [62]. The upregulation of NLRP7 in the decidual stromal cells of human first-trimester endometrium is implicated in the decidualization of endometrial stromal cells, which contributes to embryo implantation and proper placental development [63]. Decidual macrophages of the first-trimester pregnancy express the NLRP7 gene, which is related to the maintenance of endometrial hemostasis and reproductive success in humans [64]. Proinflammatory stimuli induce the expression of NLRP7 transcription in the thymus, which has effects on the inflammation and host defense [65]. In this study, early pregnancy improved the expression of NLRP7 mRNA and protein with a peak at day 13 of pregnancy, and NLRP7 protein was located in epithelial reticular cells, capillaries and thymic corpuscles. Therefore, the peak of NLRP7 at day 13 of pregnancy may be helpful for the initiation of embryo implantation, and downregulation of NLRP7 at 16 and 25 of pregnancy may be associated with the maternal immune tolerance in ewes.

## 5. Conclusions

Early pregnancy enhanced expression of NOD2 and CIITA in the maternal thymus, and NOD2, CIITA, NAIP, NLRP1, NLRP3 and NLRP7 peaked at day 13 of pregnancy. Expression of NOD1 was changed with the pregnancy stages. Furthermore, early pregnancy altered the staining intensities for NOD2 and NLRP7 in epithelial reticular cells, capillaries and thymic corpuscles. In summary, early pregnancy modulated the expression of the NLR family in the maternal thymus, which may be beneficial for the regulation of maternal thymic immune responses and pregnancy establishment during early pregnancy in sheep.

## Figures and Tables

**Table 1:** Primers used for RT-qPCR.

Gene	Primer	Sequence	Size (bp)	Accession Numbers
*NOD1*	Forward	CCTTGGCTGTCAGAGATTGGCTTC	94	XM_042248630.1
	Reverse	GCTTCTGGCTGTATCTGCTCACTG		
*NOD2*	Forward	TGCCATCCTCGCTCAGACATCTC	117	XM_042231601.1
	Reverse	CAGCCACACTGCCCTCTTTGC		
*CIITA*	Forward	GCACCTCCTTCCAGTTCCTTGTTG	119	XM_042239890.1
	Reverse	CCTGTCCCAGTCCCTGAGATCG		
*NAIP*	Forward	TTGTCCAGCAGTGTCAGCATCTTC	82	XM_012096791.3
	Reverse	ATTTCCACCACGCTGTCATCATCC		
*NLRP1*	Forward	AAGGAGGTGACCGAGATGCTGAG	143	XM_012185551.4
	Reverse	TGCCGCTTGAGTGAGGATGTATTG		
*NLRP3*	Forward	CTCTGGTTGGTCAGTTGCTGTCTC	81	XM_042250402.1
	Reverse	GGTCAGGGAATGGTTGGTGCTTAG		
*NLRP7*	Forward	GCCTGCTACTCGTTCATCCATCTC	90	XM_004015893.5
	Reverse	CCCTTCCTCCTCCTGCTCTTCC		
*GAPDH*	Forward	GGGTCATCATCTCTGCACCT	176	NM_001190390.1
	Reverse	GGTCATAAGTCCCTCCACGA		

## Data Availability

Not applicable.
